# Phenotypic study of humanized mice carrying the PAH deep intronic variant c.1199+502A>T

**DOI:** 10.1186/s13023-025-03800-6

**Published:** 2025-05-26

**Authors:** Chuan Zhang, Yousheng Yan, Bingbo Zhou, Huimin Gao, Xiaohua Jin, Ling Hui, Shengju Hao, Zongfu Cao, Xu Ma

**Affiliations:** 1 Gansu Province Medical Genetics Center, Gansu Provincial Maternity and Child-Care Hospital, Gansu Provincial Central Hospital, Lanzhou, China; 2 National Research Institute for Family Planning, National Human Genetic Resources Center, Beijing, China; 3 Graduate School of Peking Union Medical College, Beijing, China; 4 Department of Prenatal Diagnosis, Beijing Obstetrics and Gynecology Hospital, Beijing Maternal and Child Health Care Hospital, Capital Medical University, Beijing, China

**Keywords:** Phenylketonuria, Humanized mice, Phenotypic, CRISPR/Cas9, Deep intronic variant

## Abstract

**Background:**

The c.1199 + 502 A > T variant of the phenylalanine hydroxylase (*PAH*) gene, which is the most frequently reported deep intronic variant involved in phenylketonuria (PKU), is mainly observed in patients with classical or mild PKU. Prior to this study, no mouse models of PKU featuring deep intronic variants of *PAH* had been reported.

**Methods:**

To phenotypically simulate the pathogenicity of this variant, we used CRISPR/Cas9 genome editing technology and homologous recombination to generate homozygous PKU model mice with a partially humanized *Pah* gene incorporating human *PAH* exons 11–12 carrying c.1199 + 502 A > T or wild-type (c.1199 + 502WT) control sequences.

**Results:**

Humanized homozygous *Pah* c.1199 + 502 A > T mice exhibited a classical PKU phenotype, including a higher serum phenylalanine concentration, yellowing of the fur, and other traits. The homozygous mutant group had poorer spatial learning and spatial memory compared with the wild-type group.

**Conclusion:**

This construction of the first humanized mice carrying a deep intronic variant of *PAH* provides a new animal model for the pathogenesis and treatment of PKU, and may serve as a reference for future research on the pathogenicity of deep intronic variation.

## Introduction

Hyperphenylalaninemia (HPA) is the most common hereditary disorder of amino acid metabolism world-wide. The main type of HPA is phenylketonuria (PKU), which is caused by homozygous (HO) or compound heterozygous (HE) variants in the Phenylalanine hydroxylase (PAH) gene on chromosome 12q23 [1]. Although PKU has a global prevalence of approximately one in 10,000 live births [2], its occurrence varies significantly across different ethnicities and geographic regions [1]. The average incidence of PKU in China is approximately one in 15,924 people [3], with Gansu Province having the highest rate at one in 3,420 [4]. Based on blood phenylalanine (Phe) concentrations, PKU is typically classifed into three subtypes: mild HPA (MHP, 120–360 µmol/L); mild PKU (mPKU, 360–1200 µmol/L), and classic PKU (cPKU, ≥ 1200 µmol/L). While patients with mPKU and cPKU require dietary therapy, those with MHP typically do not need treatment but are advised to undergo regular monitoring [5, 6]. Untreated PKU patients can develop global developmental delay or severe irreversible intellectual disability, as well as growth failure, hypopigmentation, motor defcits, ataxia, and seizures [7]. Low rates of PKU screening and treatment negatively impact the quality of life of affected individuals and infant mortality rate [8, 9].

Many new treatments for PKU have become available in the last few years. Among these are synthetic formulations of BH4, which allows a less restrictive or non-restrictive diet in patients with mild and moderate PKU [10, 11]. Other treatment options are in preclinical or clinical development, such as mRNA, engineered probiotics and gene editing therapies [12, 13, 14]. Notwithstanding the considerable body of accumulated knowledge on PKU, a deeper understanding of its mechanisms and pathophysiology is needed. Additionally, the development of novel therapies would greatly benefit from the availability of effective model organisms [2].

The small size, high reproductive rate, and easy genetic manipulation of rodents have made mouse models a powerful research tool for preclinical testing of novel treatments. Several mouse models have been generated to study PKU pathophysiology.The first models were obtained by N-ethyl-Nnitrosourea germline mutagenesis, with Enu1 mice representing a mild HPA phenotype and Enu2 and Enu3 serving as models for severe, classical PKU [15, 16]. Because the mutations present in these mice do not correspond to human variants, more accurate models were needed to study the function of human *PAH* variants. With the advent of gene-editing technologies, mouse models carrying specific variants identified in human PKU patients, namely p.R261Q, p.R408W, p.P281L, and c.1066-11G > A, have now been generated [2, 17, 18, 19].

In recent years, an increasing number of pathogenic deep intronic variants (located > 100 bp from exon–intron boundaries) have been discovered. Among these, the c.1199 + 502 A > T variant of *PAH* is the most frequently reported [20, 21, 22]. Because c.1199 + 502 A > T has mainly been observed in patients with cPKU or mPKU [21–22], we speculated that it may contribute to these subtypes; however, no mouse models carrying deep intronic *PAH* variants have been reported. Therefore, to verify the phenotype caused by c.1199 + 502 A > T, we used CRISPR/Cas9 genome editing technology to generate a PKU mouse model with a partially humanized *Pah* gene incorporating exons 11–12 carrying the variant.

## Methods

### Generation of humanized *Pah*-c.1199 + 502 A > T knock-in mice

**Fig. 1:**
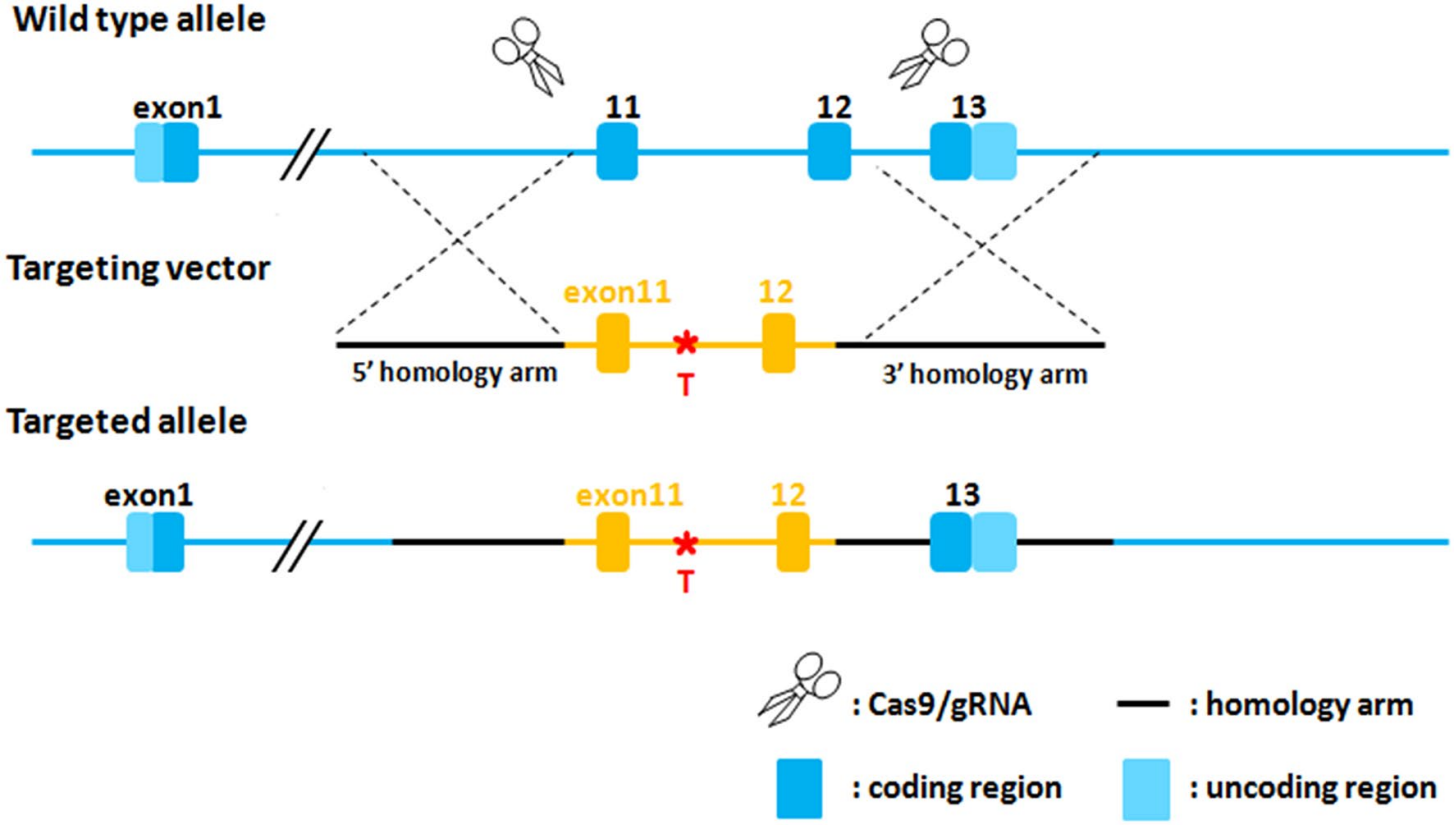
The design strategy of humanized Pah-c.1199 + 502 A > T knock-in mice

**Fig. 2:**
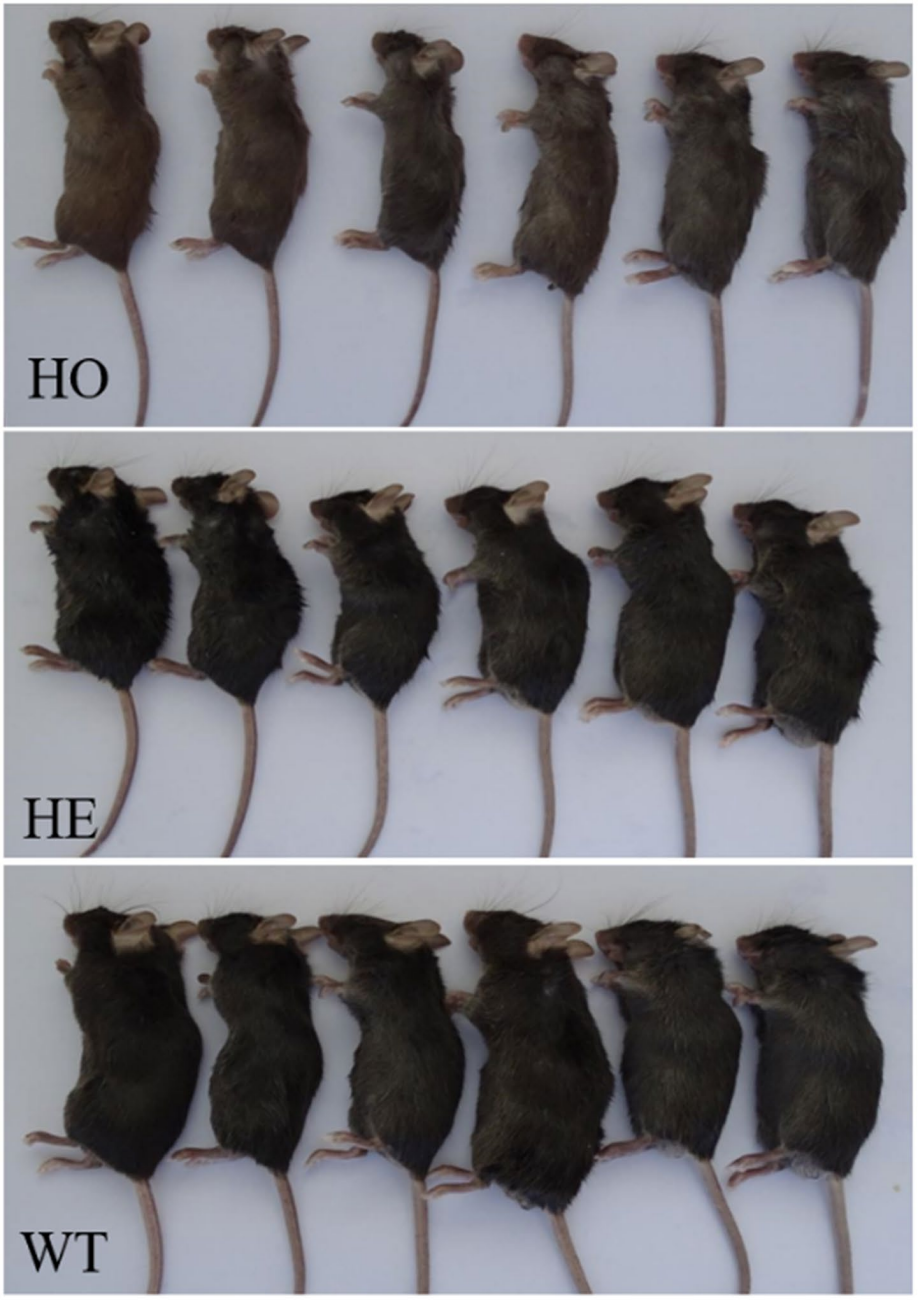
Comparison of hair color in mice with three genotypes of humanized Pah c.1199+502AT. Homozygous variant mice (HO) have poor reaction ability, weak body and yellow hair, while heterozygous mice (HE) and wild-type mice (WT) have black hair color and normal color

Using CRISPR/Cas9 technology, we introduced a knock-in of an expression box containing human *PAH* exons 11–12 with either the c.1199 + 502A > T variant or the wild-type (WT) sequence (c.1199 + 502WT) into the exon 11–12 area of the *Pah* gene of C57BL/6J mice via homologous recombination. The design strategy is shown in Fig. 1. Cas9 mRNA and guide RNA (gRNA) were synthesized via in vitro transcription. A donor vector was constructed using In-Fusion cloning, incorporating a 3.2-kb 5’ homologous arm, *PAH* exons 11–12 (c.1199 + 502A > T or c.1199 + 502WT), and a 2.7-kb 3’ homologous arm. Cas9 mRNA, gRNA, and donor vector were microinjected into the fertilized eggs of C57BL/6J mice to obtain F0 generation mice. PCR amplification and sequencing identified positive F0 generation mice, which were mated with C57BL/6J mice to obtain positive F1 generation mice (Fig. 2). The gRNA sequences were as follows: gRNA1, 5′-TGTAGTCTTAGGTGAGCCTTGGG-3′; gRNA2, 5′-AAGCTGATTCGTGTTGCTAGAGG-3′. The primers used to detect homologous recombination in F0 and F1 mice were as follows: I:5′-AGTGGCACTTCATCTCCAGC-3′; II:5′-CAGAGGGCCAACAGTCTCAG-3′; III:5′-CTTACGAGGCCACTCGGTT-3′; IV:5′-CCCAAGCAAAAGGGCAGGTA-3′.

### Mouse husbandry and colony expansion

C57BL/6J WT and transgenic mice were bred and maintained by the Shanghai Model Organisms Center, Inc. The mouse experiments were conducted in strict accordance with the relevant regulations of the Animal Experiment Management Committee. The mice were kept in a constant temperature and humidity, with dry bedding, good ventilation, and sufficient light (12-h light/dark cycle), and were given adequate water and food. All technicians conducting experiments received training in the handling of experimental animals and obtained the necessary certification for animal operation.

The colony was continuously backcrossed up to F10 with WT mice (C57BL/6J) to avoid genomic drift, in accordance with previous reports [2, 19]. HE mice were bred to produce HO mice carrying mutant *Pah* c.1199 + 502 A > T or WT *Pah* (c.1199 + 502WT), which were used in experiments. All experimental mice were adults (3–5 months-old), comprising males and females. Body weight was monitored every week.

### Genotyping

Tail DNA was extracted using the TIANGEN Blood and Tissue kit (Cat# DP304, China) in accordance with the manufacturer’s instructions. The tail DNA was amplified by standard PCR using the following primers: forward, 5’- CACCTGGTCCCTGTTCAACG-3’, and the reverse primer is 5’-CCAACAGTCTCAGAGTCCCA-3’. The amplification was carried out in a reaction system comprising 2× PCR Mix (12.5 µL), ddH20 (10 µL), DNA template (1.5 µL), and upstream and downstream primers (0.5 µL each) under the following conditions: 96 °C for 3 min; 20 cycles of 94 °C for 30 s, 60 °C for 30 s, and 72 °C for 1 min; 15 cycles of 94 °C for 30 s, 60 °C for 30 s, and 72 °C for 1 min; 72 °C for 10 min; and storage at 4 °C. The PCR amplification products were purified with purification kits(TIANGEN, Cat# DP204, China), sequenced on an ABI 3500DX Genetic Analyzer (Applied Biosystems, Foster City, CA, USA), and compared with standard reference sequences using SeqMan software (v 7.1.0).

### Analysis of amino acid metabolism

One drop of blood collected from the mouse retro-orbital sinus was applied to a prepared blood collection card, air dried, and stored at 4 °C. Employing a 3.2-mm hole punch, disks from the dried blood spot were analyzed using liquid chromatography-tandem mass spectrometry (LC-MS/MS) on a TQD system (Waters, USA).

### Metabolic cage

Following an acclimatization period of 8–12 h, physiological parameters (rates of O_2_ consumption and CO_2_ production, food intake, activity) were directly measured in 3- to 4-month-old humanized *Pah* c.1199 + 502 A > T and control WT mice (*n* = 4 mice per group) using the Oxymax Comprehensive Lab Animal Monitoring System (CLAMS; ). Data were recorded over a 72-h period. Other calorimetric properties, including respiratory exchange rate and energy expenditure, were indirectly calculated using the classical equations developed by Paul Lusk, as provided in the Oxymax processing software.

### Morris water maze experiment

The Morris water maze experiment was carried out in a circular pool (1.2-m diameter) that was divided into four quadrants using a vertical cross. Black markers of different shapes were affixed to the light-colored pool walls to distinguish each quadrant. The circular escape platform (diameter: 9 cm) was placed in the water at the center of the I quadrant (target quadrant). The pool was surrounded by a curtain to separate the experimenter and the animal subject, preventing the animal from using the experimenter as a visual location marker. Before starting the experiment, the pool was filled with tap water to a depth of ~ 25 cm, and the water temperature was adjusted to 23 ± 1 °C. To prevent the mice from seeing the underwater escape platform, titanium dioxide was added to the water until it turned a milky white color. At the end of each training session, the mice were removed from the platform, dried with a towel, and returned to normal body temperature under warm air flow. Spatial learning and memory were evaluated via escape latency, time spent in the target quadrant, and frequency of crossing the platform.

### Statistical analysis

Quantitative data are presented as mean ± standard deviation (SD). Statistically significant differences between pairs of groups were determined by unpaired two-tailed t-tests. For comparisons of the three experimental groups, one-way analysis of variance (ANOVA) was used. Throughout the study, the following critical values were used to determine statistical significance: *P < 0.05, **P < 0.01, ***P < 0.001, and ****P < 0.0001.

## Results

### PKU phenotype in humanized *Pah* c.1199 + 502 A > T model mice

Mice carrying humanized WT *Pah* and HO or HE c.1199 + 502 A > T *Pah* variant alleles were selected from the F2 generation mice and monitored for reaction ability, hair color, growth, and development. HO mice exhibited poor responsiveness, body weakness, yellowing of the fur (Fig. 2), and initial symptoms of PKU. At 4 weeks, the body weight of HO mice was significantly lower than that of HE (*P* < 0.001) and WT (*P* < 0.001) mice (Table 1). There was no significant difference in body weight between WT mice and HE mice (*p* = 0.807).

**Table 1:** Weight comparison results of three genotypes of mice

Genotype	Weight Range (g)	X ± 2SD(g)	t	*p*
HO(n = 8)	8.0-13.6	10.33 ± 1.74		
HE(n = 25)	9.5–20.5	15.87 ± 3.37	-5.990	< 0.001*
WT(n = 13)	9.9–20.2	15.69 ± 3.20	-4.852	< 0.001^#^

HO: homozygous mutant Pah c.1199 + 502A > T, WT: homozygous wild-type (c.1199 + 502WT), HE: heterozygous.* Comparison of HO mutant mice with HE mice, # comparison of HO mutant mice with WT (c.1199 + 502WT)

### LC-MS/MS analysis of humanized *Pah* c.1199 + 502 A > T model mice

Next, we performed LC-MS/MS analysis of the serum from HO, HE, WT, and normal C57BL/6J mice. As shown in Table 2, t-tests showed that the serum Phe content of HO mutant mice was higher than that of HE (*P* < 0.001) and WT (*P* < 0.001) mice, with no significant difference between HE and WT mice (*P* = 0.065).

**Table 2:** Comparison of blood phe concentration in mice

Group	Genotype	Phe range (µmol/L)	X ± 2SD(µmol/L)	t	*p*
1	HO (n = 9)	1093.63-1904.35	1394.14 ± 272.42	14.65	< 0.001
	HE (n = 26)	35.73–91.89	63.00 ± 16.94		
2	HO (n = 9)	1093.63-1904.35	1394.14 ± 272.42	14.756	< 0.001
	WT (n = 15)	30.03–78.72	53.22 ± 13.74		
3	HE(n = 26)	35.73–91.89	63.00 ± 16.94	1.902	0.065
	WT(n = 15)	30.03–78.72	53.22 ± 13.74		

HO: homozygous Pah c.1199 + 502AT, WT: homozygous wild-type (c.1199 + 502WT), HE: heterozygous

### Effects of the humanized *Pah* c.1199 + 502 A > T variant on metabolic parameters

**Fig. 3:**
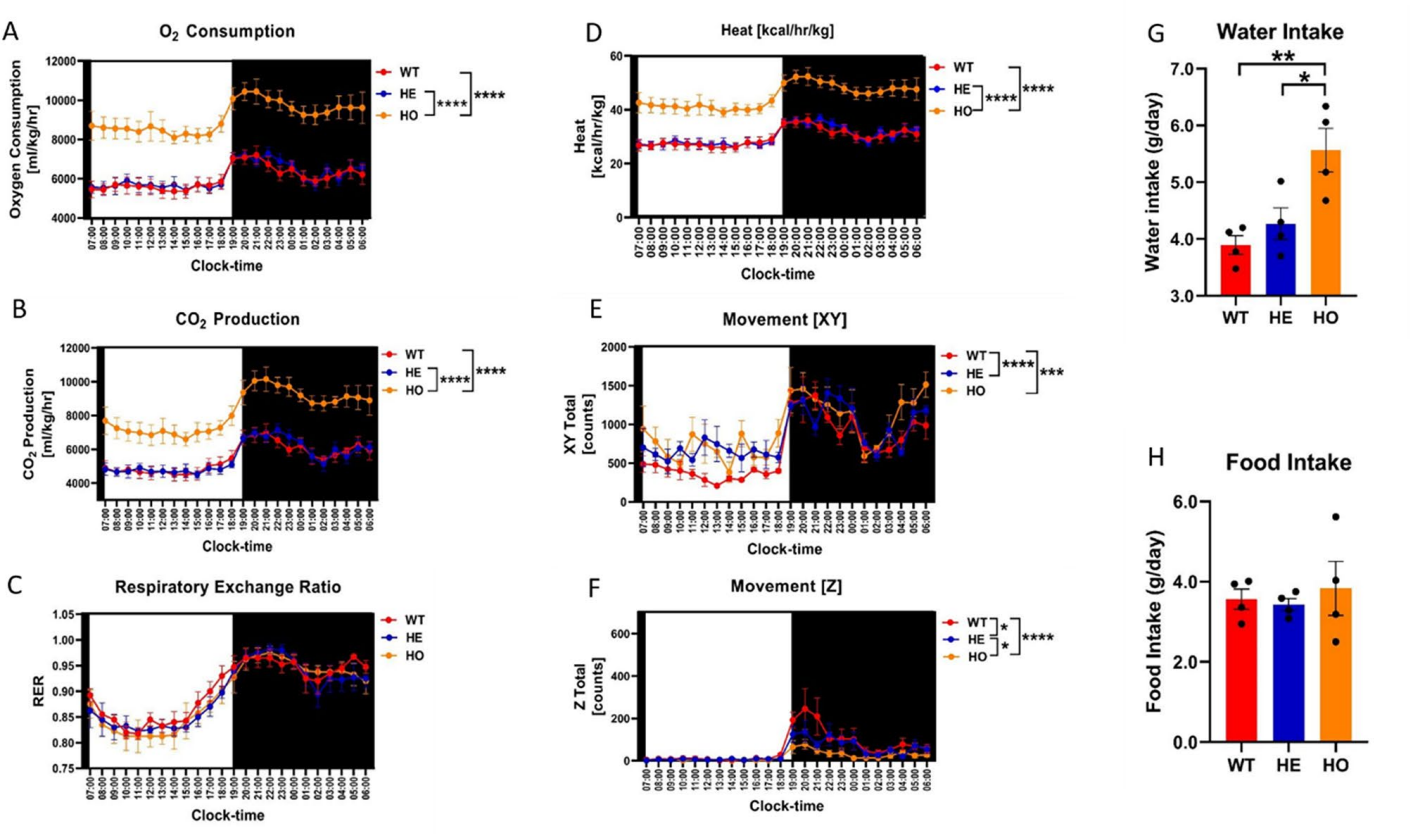
Physiological and metabolic characterization of three genotype mice. **A**: Oxygen consumption in HO group (n=4) was significantly higher than that in WT group (n=4) and HE group (n=4) (two-way ANOVA, ****, *p* < 0.0001), **B**: CO2 production in HO group (n=4) was significantly higher than that in WT group (n=4) and HE group (n=4) (two-way ANOVA, ****, *p* < 0.0001), **C**: There was no significant difference in respiratory exchange rate among the three groups (two-way ANOVA, n.s.), **D**: Caloric production in HO group (n=4) was significantly higher than that in WT group (n=4) and HE group (n=4) (two-way ANOVA, ****, *p* < 0.0001), **E**: The horizontal movement of mice in HO group (n=4) and HE group (n=4) was significantly higher than that in WT group (n=4) (two-way ANOVA, ***, *p* < 0.001, ****, *p* < 0.0001), **F**: The vertical movement in HO group (n=4) was significantly lower than that in WT group (n=4) and HE group (n=4). The vertical movement amount of mice in HE group (n=4) was significantly lower than that in WT group (n=4) (two-way ANOVA, *, *p* < 0.05, ****, *p* < 0.0001), **G**: The average daily water intake of HO group (n=4) was significantly higher than that of WT group (n=4) and HE group (n=4) (Unpaired t-test, *, *p* < 0.05, **, *p* < 0.01), **H**: There was no significant difference in the average daily food intake of the groups (Unpaired t-test, n.s.)

Oxygen consumption was significantly higher in the HO group than in the WT and HE groups (*n* = 4/group; ****P < 0.0001 by two-way ANOVA; Fig. 3). CO_2_ production was significantly higher in the HO group than in the WT and HE groups (*n* = 4/group; ****P < 0.0001 by two-way ANOVA). There were no significant differences in the respiratory exchange rate among the three groups (two-way ANOVA). Caloric production was significantly higher in the HO group than in the WT and HE groups (*n* = 4/group; ****P < 0.0001 by two-way ANOVA). There was significantly more horizontal movement in the HO and HE groups than in the WT group (*n* = 4/group; ***P < 0.001 and ****P < 0.0001 by two-way ANOVA). Vertical movement was significantly reduced in the HO group compared with that in the WT and HE groups, and was significantly reduced in the HE group compared with that in the WT group (*n* = 4/group; *P < 0.05 and ****P < 0.0001 by two-way ANOVA). While the average daily water intake was significantly higher in the HO group than in the WT and HE groups (*n* = 4/group; *P < 0.05 and **P < 0.01 by an unpaired t-test), there were no significant between-group differences in the average daily food intake, as determined by an unpaired t-test (Fig. 3).

### Effects of the humanized *Pah* c.1199 + 502 A > T variant on spatial learning and memory

**Fig. 4:**
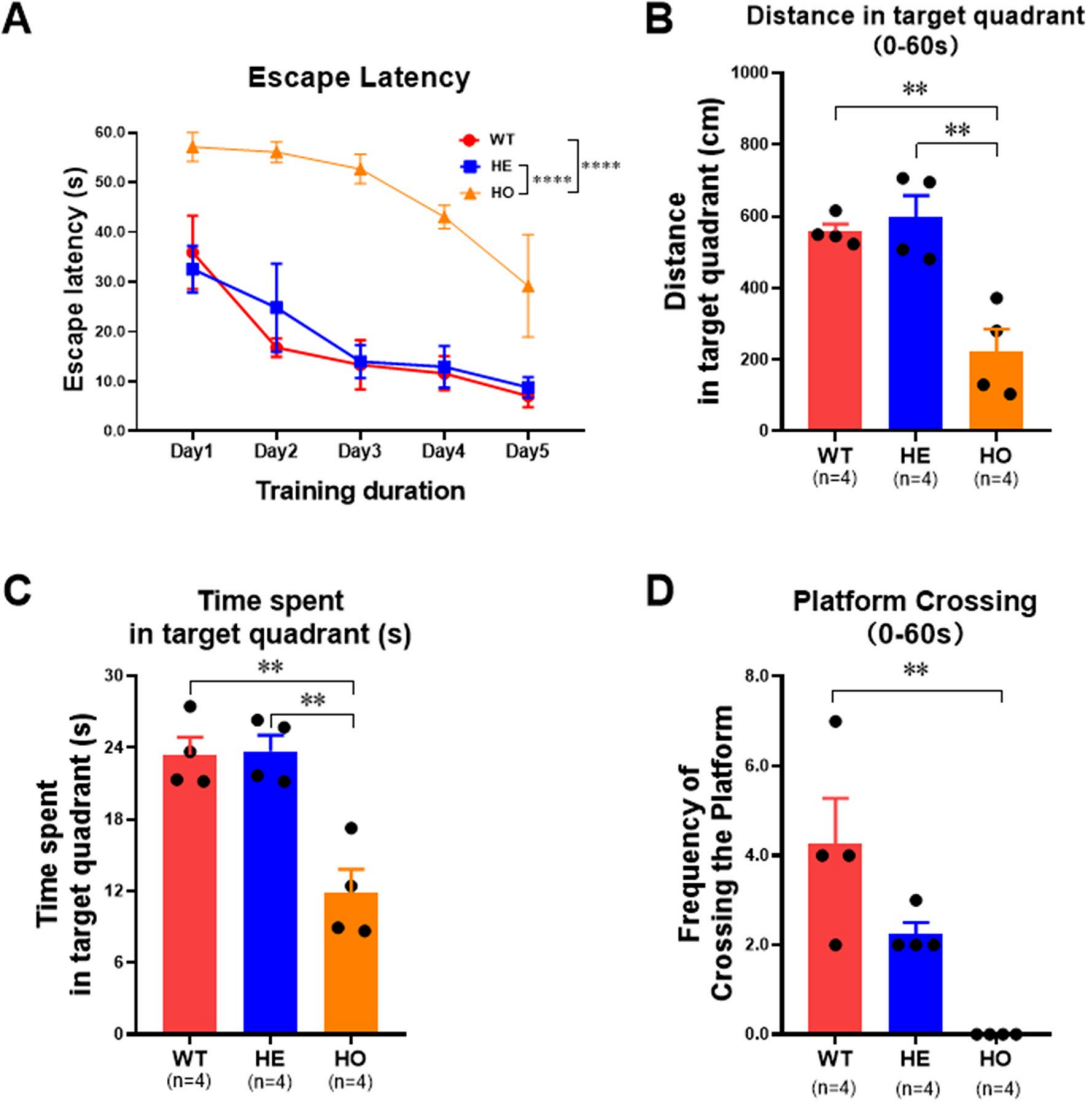
The Escape Latency, Time spent in target quadrant and Frequency of crossing the platform in Morris water maze experiment were used to evaluate the spatial memory ability of the animals.The results of Morris water maze experiment showed that the Escape Latency of mice in HO group (n=4) was significantly higher than that in HE group (n=4) and WT group (n=4) during the training period (two-way ANOVA, ****, *p* < 0.0001). The experimental results of the test period showed that mice in HO group (n=4), compared with mice in HE group (n=4) and WT group (n=4), Distance in target quadrant(0-60s) and Time spent in target quadrant(0-60s) were significantly reduced. The Platform Crossing(0-60s) in HO group (n=4) was significantly lower than that in WT group (n=4) (Odinary one-way ANOVA, **, *p* < 0.01)

Escape latency, time spent in the target quadrant, and frequency of crossing the platform in the Morris water maze experiment were used to evaluate spatial learning and memory in the mice (Fig. 4). Escape latency during the training period was significantly longer in the HO group than in the HE and WT groups (*n* = 4/group; ****P < 0.0001 by two-way ANOVA). Using a 60-s test period, distance traveled and time spent in the target quadrant were significantly reduced in the HO group compared with those in the HE and WT groups, while the frequency of platform crossing was significantly lower in the HO group than in the WT group (**P < 0.01 by ordinary one-way ANOVA; Fig. 4).

## Disscusion

The mouse model is a powerful research tool, particularly for studying human diseases, because of its small size, high reproductive rate, and relative ease of genetic manipulation, which set it apart from other mammals [23]. Although mice and humans differ significantly in terms of evolutionary factors, such as body size, metabolic rate, life expectancy, and immune systems, they share a high degree of genetic and physiological similarity overall [23].

Three first-generation HPA/PKU mouse models were created through phenotypically driven germline mutagenesis using N-ethyl-N-nitrosourea [2]: (1) Enu1, which carries the p.V106A variant located in the *Pah* regulatory domain [15]; (2) Enu2, which carries the p.F263S variant located in the catalytic domain sequence of *Pah* [15]; and (3) Enu3, which has splice site variants that produce frameshift amino acids and early termination codons in *Pah* [16]. Enu2 and Enu3 mice exhibit high blood concentrations of Phe, and are often regarded as severe typical PKU models. Enu2 mice have normal *Pah* gene stability, but the protein it produces is completely inactive, while Enu3 mice do not exhibit any *Pah* activity or protein [16]. Enu 1 mice have decreased *Pah* stability, reducing PAH protein stability and enzymatic activity to about 5% of normal controls, thereby resulting in mild HPA [24]. Mouse models have aided in elucidating PKU at the biochemical and behavioral levels, testing new treatments (e.g., enzyme replacement therapy and gene therapy) [25, 26], and mimicking the phenotypes of human *PAH* variants.

In 2021, Aubi et al. [2] reported the first construction of a mouse model with a point mutation of *Pah*. They applied CRISPR/Cas9 technology to introduce the human *PAH* gene mutation c.782G > A (R261Q) into the mouse genome, resulting in mice with an MHP phenotype. However, patients with HO variants of *PAH* c.782G > A(R261Q) exhibit mPKU and cPKU [27, 28]. The *PAH* variant c.1066-11G > A, which is the second most prevalent (2.6%) genotype worldwide and the most prevalent in Southern Europe, is associated with the cPKU phenotype [19]. In 2024, Martínez-Pizarro et al. [19] used CRISPR/Cas9 to generate humanized c.1066-11G > A *PAH* mice that exhibit a cPKU phenotype. These findings suggest that genotypic and phenotypic associations of PAH may differ between humans and mice.

The c.1199 + 502 A > T *PAH* mutation is the most frequently reported deep intronic variant [20, 21, 22] and is classified as a pathogenic variant (PS3 + PM3_strong + PP4) in the American College of Medical Genetics and Genomics guidelines. Evidence supporting this classification includes the PS3 criterion, for which *PAH* minigene experiments and reverse transcription PCR of blood RNA from a PKU patient confirmed that the c.1199 + 502 A > T variant may strengthen the predicted branch point and lead to the inclusion of a 25-nt pseudo-exon in the *PAH* mRNA [20]. Additionally, the PM3_strong criterion was met in > 18 patients with PKU, in whom the c.1199 + 502 A > T variant was identified in *trans* with a known pathogenic variant. Finally, the PP4 criterion has been satisfied by patients with a phenotype highly specific for PKU. Because the c.1199 + 502 A > T variant is mainly observed in patients with cPKU or mPKU [21–22]; we speculated that it might cause these conditions. To verify this, we used CRISPR/Cas9 genome editing technology to generate a PKU mouse model with a partially humanized *Pah* intron 11 carrying the variant.

The blood Phe level of our HO humanized *Pah* c.1199 + 502 A > T mice (1394.14 ± 272.42 µmol/L) was higher than that of the HO *Pah* c.782G > A(R261Q) model mice constructed by Aubi et al. [2] (108.0 ± 36.6 µmol/L), and similar to that of the mutant mouse model constructed by Martinez-Pizarro et al. (1885.7 ± 71.2 µmol/L) [19]. This demonstrated that the blood profile of our HO c.1199 + 502 A > T mutant mice was consistent with the cPKU phenotype. Consistently, the blood Phe levels of our HO *Pah* c.1199 + 502 A > T WT mice (53.22 ± 13.74 µmol/L) and the WT mice in the Aubi et al. study (59.9 ± 7.7 µmol/L)^2^ were similar.

The impact of PKU on growth is still debated. Although some studies have reported that PKU is associated with reduced birth weight and length of affected infants [29, 30], a recent meta-analysis showed that fetal growth in children with PKU is typically normal [31]. One study showed that, compared with a reference population, children with cPKU grew normally at birth and in infancy, but were shorter and weighed less in the first 4 years of life. By contrast, patients with mPKU did not exhibit growth disorders and did not need to restrict their diet [31]. A study on the height and growth of PKU patients from birth to adulthood in Germany showed that PKU patients are significantly shorter than healthy controls, by 3 cm in females and 5 cm in males, with significantly reduced growth rates during the first 2 years of life and adolescence [32]. Results of a Slovenian study showed that untreated cPKU patients tend to be shorter in adulthood and that a low-protein diet may delay growth and development in childhood. By contrast, treated cPKU patients typically achieve normal adult height, while benign MHP has no effect on height or weight [33].

Enu2 model mice have a shorter body length and lighter body weight than WT mice [34] In mutant mice carrying HO *Pah* c.782G > A(R261Q), there was no statistical difference in body weight compared with WT mice [2] In our study, the body weight of HO *Pah* c.1199 + 502 A > T mice was significantly lower than that of WT and HE mice. Additionally, we inferred that our HO *Pah* c.1199 + 502 A > T mice and Enu2 mice share the same cPKU phenotype, and that HO *Pah* c.782G > A(R261Q) mice exhibit an MHP phenotype of no growth retardation.

In our study, the amount of food intake did not significantly differ between HO *Pah* c.1199 + 502 A > T mutant and WT mice, consistent with the similar intake reported for HO *Pah* c.782G > A(R261Q) mutant and WT mice [2]. However, the average daily water intake of our HO *Pah* c.1199 + 502 A > T group was significantly higher than that of the HE *Pah* c.1199 + 502 A > T and WT groups. The rates of O_2_ consumption and CO_2_ production were reportedly lower in HO *Pah* c.782G > A(R261Q) mutant mice compared with those in WT mice, but were significantly higher in our HO *Pah* c.1199 + 502 A > T group than in our HE and WT groups. There were no significant differences in activity or movement patterns between HO *Pah* c.782G > A(R261Q) and WT mice; however, the HO *Pah* c.1199 + 502 A > T group exhibited significantly more horizontal movement and significantly less vertical movement than the WT group.At present, there are no clear scientific data directly comparing the metabolic characteristics of PKU mice and normal mice in terms of food intake, daily water intake, and rates of O_2_ consumption and CO_2_ production. In PKU patients, the disease characteristics are mainly caused by enzyme defects in the Phe metabolic pathway, leading to the accumulation of Phe and its ketoacids. In mice, these metabolic abnormalities may alter physiological and overall bodily functions, potentially affecting metabolic characteristics. Differences in metabolic characteristics between the *Pah* c.782G > A(R261Q) and *Pah* c.1199 + 502 A > T variants may be attributable to the former being associated with the MHP phenotype and the latter with the cPKU phenotype. However, this is only a hypothesis and requires further validation with a larger sample size.

Untreated PKU patients can develop global developmental delay or severe irreversible intellectual disability [7]. In the present study, the escape latency during the training period in the Morris water maze experiment was significantly longer in the HO *Pah* c.1199 + 502 A > T group than in the WT group. Furthermore, mice in the HO mutant group showed significant reductions in distance traveled and time spent in the target quadrant compared with mice in the WT group. The platform crossing frequency was also in significantly lower in the HO group than in the WT group. Collectively, these results indicated that the HO *Pah* c.1199 + 502 A > T mutants had poorer spatial learning and memory compared with WT mice, consistent with cPKU phenotype.

In conclusion, this is the first report of a deep-intronic humanized *Pah* c.1199 + 502 A > T variant knock-in mouse. HO *Pah* c.1199 + 502 A > T model mice exhibited a cPKU phenotype, including higher serum Phe concentrations, retarded cognitive development, yellowing of the fur, and other symptoms. HO *Pah* c.1199 + 502 A > T mice also showed poorer spatial learning and memory compared with the WT group. Our findings support the use of this new animal model to study the pathogenesis and treatment of PKU, and provide a reference for future research on the pathogenicity of deep intronic variants.

## Data Availability

The data that support the findings of this study is available upon reasonable request from corresponding authors.

## Declarations

### Study approval

We have complied with all relevant ethical regulations for mice breeding, testing and research. The animal experiments in this study have received the appropriate from the Ethics Committee of the Gansu Provincial Maternity and Child-Care Hospital(2021GSFY[65]). The relevant experiments on mice are conducted in strict accordance with the relevant regulations of the Animal Experiment Management Committee. The experimental operators engaged in this study have been trained on experimental animals and obtained the working certificate of experimental animal operation.

## Abbreviations

cPKU: Classic PKU
HO: Homozygous
HPA: Hyperphenylalaninemia
MHP: Mild hyperphenylalaninemia
mPKU: Mild PKU
MT: Mutant
PKU: Phenylketonuria
Phe: Phenylalanine
WT: Wild type
